# The mediating role of social anxiety, loneliness and perfectionism in the relationship between fear of missing out (FoMO) and digital addiction

**DOI:** 10.1186/s40359-026-04489-3

**Published:** 2026-04-21

**Authors:** Gulsen Filazoglu Cokluk

**Affiliations:** Toros University, Mersin, Turkey

**Keywords:** FoMO, Digital Addiction, Social Anxiety, Loneliness, Perfectionism

## Abstract

**Objective:**

The primary objective of this study is to examine the mediating roles of social anxiety, loneliness, and perfectionism in the relationship between fear of missing out (FoMO) and digital addiction.

**Method:**

This study employed a quantitative correlational design to examine the mediating roles of loneliness, social anxiety, and perfectionism in the relationship between FoMO and digital addiction. The sample consisted of 2,372 young adults aged 18–30 in Turkey. Data were collected online using validated self-report scales administered through Google Forms. Analyses were conducted using SPSS 25.0 and Hayes’ PROCESS macro (Model 4) with 5,000 bootstrap samples.

**Results:**

Correlation analyses revealed that digital addiction was positively associated with social anxiety andFoMO, but negatively related to perfectionism and loneliness. Mediation analysis using PROCESS Model 4 indicated that FoMO significantly predicted digital addiction, both directly and indirectly through social anxiety. The mediating effects of loneliness and perfectionism were nonsignificant. These results suggest that individuals with higher FoMO are more prone to digital addiction, primarily due to increased social anxiety.

**Discussion:**

The research findings were discussed within a theoretical and conceptual framework, and similarities and differences in the literature were highlighted.

**Supplementary Information:**

The online version contains supplementary material available at 10.1186/s40359-026-04489-3.

## Introduction

Digital addiction refers to the excessive and uncontrolled exposure of individuals to digital devices and online content. With the widespread proliferation of the Internet and social media platforms today, there has been an increase in digital addiction. The rapidly evolving digital landscape has led to growing concern regarding the phenomenon of digital addiction and its associated psychological consequences.

### The relationship between FoMO and digital addiction

## The Relationship Between FoMO and Digital Addiction

Digital addiction encompasses a variety of compulsive behaviors involving excessive attachment to social networks, online games, and other digital platforms. This condition can manifest through symptoms such as withdrawal, the development of tolerance, and adverse effects on daily functioning [1]. One psychological construct that has garnered significant attention in this context is the Fear of Missing Out (FoMO)-a cognitive-emotional state characterized by anxiety related to the perception that others are engaging in rewarding experiences from which one is absent [2]. Although some prior literature has conceptualized FoMO as a *result* of excessive digital engagement [3], the current study reverses this directionality: FoMO is framed as a *predictor* and a motivational driver of digital addiction, primarily through its psychological effects such as social anxiety and maladaptive comparison processes [4, 5]. Individuals experiencing high levels of FoMO may feel compelled to remain constantly connected to digital environments in order to stay socially included, which increases both frequency and intensity of use, thereby facilitating addiction [6].

### The mediating role of social anxiety

FoMO denotes the constant desire to stay online due to concerns about missing out on social interactions and shared experiences. This psychological tension generates heightened anxiety, especially among younger demographic groups who are heavily engaged in digital interactions, and it may exacerbate social anxiety. This study proposes a multi-level model that examines digital addiction, FoMO and social anxiety together. The findings of the study will reveal the effects of digital addiction and FoMOwithin specific social contexts and offer new perspectives on individuals’ mental health. From this perspective, The findings are consistent with the possibility that social anxiety may function as an explanatory mechanism linking FoMO and digital addiction. as an antecedent variable that elevates vulnerability to digital addiction, particularly when mediated by social anxiety-a dynamic supported by both theoretical and empirical findings [7, 8]. The present study tests an association-based mediation model in which FoMO is positively associated with digital addiction partially through increased levels of social anxiety, while also accounting for the potential roles of loneliness and perfectionism as mediators Building on prior research examining FoMO and digital behaviors as a phenomenon that emerges when individuals constantly follow the lives of others through social media, reinforcing feelings of anxiety, inadequacy, and loneliness [2]. The current study found that participants who experience high levels of FoMO exhibit significantly increased levels of social anxiety.

### The mediating role of loneliness and maladaptive perfectionism

Individuals with perfectionist tendencies are constantly exposed to more idealized and flawless lives on social media; this, in turn, reinforces feelings of envy and inadequacy. Perfectionism is closely related to social anxiety and leads individuals to experience more intense anxiety in their social interactions [9].

From a societal perspective, developing effective strategies to cope with digital addiction is of great importance. According to the recommendations of Curran et al. [10], it is necessary to teach individuals to limit their use of digital media and to offer educational programs that promote healthy social interactions. Additionally, organizing campaigns aimed at raising public awareness constitutes a critical approach. In particular, enabling young individuals to become aware of the negative effects of digital addiction can serve as an effective mechanism in combating social anxiety, loneliness, and perfectionism. As emphasized by Lin et al. [11], programs aimed at strengthening individuals’ offline social interactions are considered effective strategies to fill the void created by digital media. Within this framework, adopting a healthy and balanced lifestyle that goes beyond mere existence in the digital world emerges as a vital necessity. Emerging adulthood is a critical developmental period marked by identity exploration, heightened sensitivity to social evaluation, and an increased need for belonging and peer approval Arnett, [40, 41]. During this stage, young adults are particularly vulnerable to social anxiety and fear of social exclusion, while simultaneously being among the most intensive users of digital and social media platforms Twenge et al., [42]. Digital environments may therefore become especially salient for individuals experiencing FoMO, as constant online connectivity provides immediate access to social information and perceived inclusion. However, excessive reliance on digital media during this period has been associated with elevated social anxiety, loneliness, and maladaptive perfectionism, particularly through social comparison processes Elhai et al., 2016; Dhir et al., [46]. Accordingly, focusing on emerging adults provides a developmentally grounded rationale for examining the interplay between FoMO, psychosocial vulnerabilities, and digital addiction.

Existing studies reveal a strong relationship between the severity of digital addiction symptoms and high levels of FoMO For instance, a study conducted by Zhu et al. [12] found that individuals exhibiting higher levels of digital addiction also reported greater FoMO. Similarly, Li et al. [8] emphasized that students who used social networks intensively showed significant increases in FoMO prevalence, particularly in competitive environments such as university campuses. This phenomenon, often characterized by phases of transition and identity formation, reflects an intersection where academic and social pressures are further intensified by digital connectedness.

### Social comparison processes and their contribution to FoMO

In fact, the tendency for social comparison is a phenomenon observed well before the digital age-emerging as an urge for active information seeking intertwined with the fear of exclusion. Today, in digital environments, FoMO has been linked to intensified social comparison processes.and can be interpreted as a continuation of previously observed behaviors of social comparison [9]. However, this need for social comparison may lead to excessive use of social media and addiction [13]. Moreover, a strong connection has been identified between tendencies for social comparison, FoMO, and addiction to social networking sites [14]. Accordingly, social comparisons based on success, wealth, and performance can give rise to intense meta-emotions such as envy, hatred, and resentment, and at this point, FoMO becomes associated with subjective well-being [9].

Previous research has addressed the complex nature of the relationship between digital addiction and social anxiety. Alt & Boniel-Nissim [4], revealed that increased time spent in digital environments elevates levels of social anxiety. His studies demonstrated that the constant need for individuals to remain online intensifies concerns related to social interaction and triggers feelings of loneliness. In addition, Efeoğlu [15] examined the impact of FoMO on social anxiety and found that the competitive environment experienced through social media negatively affects social performance. These findings are significant for understanding the psychological effects of digital addiction in relation to social anxiety.

The scope of these findings can be understood within the context of broader psychological mechanisms. Rumination-defined as the tendency to engage in repetitive negative thought patterns-and feelings of loneliness are key mediating elements in the relationship between digital addiction and FoMO [16]. Individuals with digital addiction are often reinforced by negative social feedback or perceived social failure. This cognitive process further intensifies their FoMO and creates a vicious cycle in which digital interaction becomes a coping mechanism, thereby reinforcing the addiction itself [17]. This phenomenon is particularly prominent among university students and young adults, who are simultaneously coping with academic pressures and the development of their self-identity. The dependence on digital approval may intensify feelings of exclusion and result in higher levels of social anxiety [8].

This cyclical relationship can be partially explained by social comparison theory, which posits that individuals assess their social status by comparing themselves to their peers. In the digital environment, platforms that focus on presenting carefully curated moments of life compel individuals into a constant comparison process, thereby increasing feelings of inadequacy and loneliness when social activities or trends are missed [9].

### Demographic and cultural context in FoMO–digital addiction dynamics

Social influences also play a critical role in shaping these dynamics. The emergence of digital platforms has normalized constant online presence, creating an environment in which social approval is increasingly measured through likes, comments, and shares. The pervasive nature of social networks fosters a culture of comparison and fear, particularly when users are prone to perfectionism or are more sensitive to social cues [18]. These social pressures do not affect everyone equally; research has shown that demographic factors such as age, gender, and socioeconomic status can determine how FoMO and social anxiety manifest and relate to one another. For instance, it has been found that young women exhibit higher levels of FoMO, which may stem from societal conditioning centered around social relationships and physical appearance [19].

At this point, it is useful to consider the concept of perfectionism. Perfectionism is associated with both a strong desire for achievement and a fear of social rejection [20, 21], and it increases the tendency for social comparison [22]. Maladaptive perfectionism produces negative effects such as self-criticism and a focus on mistakes [23], and it is linked to social media burnout [24]. Its relationship with FoMO has been limitedly explored; however, it has been shown that maladaptive perfectionism reinforces digital hoarding behavior [25].

Demographic characteristics such as age and gender are described descriptively in the present study, Particularly, individuals raised in collectivist cultural settings may experience higher levels of social anxiety due to community expectations and relational dynamics; this situation creates a fertile ground for intensified experiences of FoMO during interactions with digital platforms that reflect idealized forms of social life [16]. In this context, it is important to develop a more nuanced and multilayered perspective on how demographic variables interact with psychological processes. The complex nature of digital addiction, the triggering role of FoMO, and their potential effects on social anxiety constitute a significant area of research in the current literature [8].

Social factors also play a critical role in shaping these dynamics. The widespread adoption of digital platforms has normalized constant online presence and transformed social approval into quantifiable metrics such as “likes” and comments. This environment fosters a culture of comparison and sets the stage for the entrenchment of FoMO, particularly among individuals with a tendency toward perfectionism or heightened sensitivity to social cues [26]. Moreover, cross-cultural findings indicate that collectivist cultural contexts may strengthen the link between digital addiction, FoMO, and social anxiety by increasing social anxiety and related behaviors [18, 27]. Therefore, demographic variables such as age, gender, and cultural background may shape levels of FoMO and related psychological processes, although they are not examined as moderators in the present study. This highlights the necessity of culturally sensitive research and interventions aimed at understanding digital-based psychopathology.

Self-Determination Theory (SDT) provides a robust theoretical framework for understanding vulnerability to problematic digital engagement by emphasizing the role of basic psychological needs, namely autonomy, competence, and relatedness Deci & Ryan, [7] Ryan & Deci, (2017). In particular, frustration of the need for relatedness has been shown to heighten individuals’ sensitivity to social evaluation, exclusion, and belonging-related concerns, thereby increasing reliance on external sources of validation (Vansteenkiste et al., 2020). Within contemporary digital environments, FoMO reflects unmet relatedness needs and concerns about social exclusions Przybylski et al., [2]. From an SDT perspective, FoMO can be conceptualized as a motivational signal reflecting deficits in need satisfaction, particularly relatedness, which may drive individuals toward excessive engagement with social media and digital platforms in an attempt to restore social connection and reassurance (Milyavskaya et al., 2018; Sheldon & Gunz, 2009). However, this compensatory digital engagement may also intensify social-evaluative concerns and interpersonal self-monitoring, thereby increasing levels of social anxiety and reinforcing maladaptive patterns of use Elhai et al., 2016; Wegmann et al., [45]. Accordingly, social anxiety is theoretically positioned as a proximal psychological mechanism through which FoMO translates into digital addiction. Although loneliness and maladaptive perfectionism are also conceptually linked to unmet psychological needs and social concerns, existing evidence suggests that these variables may function as more distal or contextual vulnerabilities rather than primary mediating mechanisms in this pathway Casale et al., 2014; Burnell et al., [47]. Grounded in this integrative framework, the present study combines SDT with cognitive–behavioral perspectives to examine whether social anxiety, loneliness, and perfectionism mediate the relationship between FoMO and digital addiction among young adults. Given the cross-sectional design of the present study, the proposed model reflects statistical associations rather than causal or temporal relationships.

### Research question

- RQ1: Do social anxiety, loneliness, and perfectionism mediate the association between FoMO and digital addiction?
- RQ1a: Does social anxiety significantly mediate the relationship between FoMO and digital addiction?
- RQ1b: Does loneliness significantly mediate the relationship between FoMO and digital addiction?
- RQ1c: Does perfectionism significantly mediate the relationship between FoMO and digital addiction?

Drawing on Self-Determination Theory and cognitive–behavioral models, this study conceptualizes fear of missing out (FoMO) as a motivational antecedent of digital addiction that operates both directly and indirectly through psychosocial mechanisms. Based on this framework, the following hypotheses were formulated to test the mediating roles of social anxiety, loneliness, and perfectionism.

### Hypotheses

Hypothesis.

- H1: FoMO is positively associated with digital addiction.
- H2: FoMO is positively associated with social anxiety.
- H3: Social anxiety mediates the relationship between FoMO and digital addiction.
- H4: Loneliness and maladaptive perfectionism are tested as alternative mediators in the relationship between FoMO and digital addiction.

Despite growing evidence linking FoMO to digital addiction, several gaps remain in the literature. First, previous studies have primarily examined bivariate associations rather than testing integrated mediation models. Second, the simultaneous roles of social anxiety, loneliness, and maladaptive perfectionism have rarely been investigated within a unified structural framework. Third, limited research has explored these mechanisms in non-Western cultural contexts. Addressing these gaps may provide a more comprehensive understanding of the psychological processes underlying digital addiction. In light of these gaps, the present study proposes building upon the aforementioned findings, the present study examines the roles of digital addiction, FoMO, social anxiety, perfectionism, and loneliness in a sample consisting of young adults aged between 18 and 30.Accordingly, the primary aim of this research is to investigate the associations between digital addiction, FoMO, and social anxiety by examining the mediating roles of loneliness and maladaptive perfectionism. These psychological factors are considered central mechanisms that may help explain how FoMO and digital addiction relate to social anxiety. In particular, loneliness may increase individuals’ orientation toward digital environments, while maladaptive perfectionism may intensify self-evaluative concerns within social contexts, thereby contributing to heightened vulnerability in digital engagement patterns.

### Method

This section of the study provides information regarding the research model, study group, data collection instruments, procedure, and data analysis.

### Research model

**Fig. 1:**
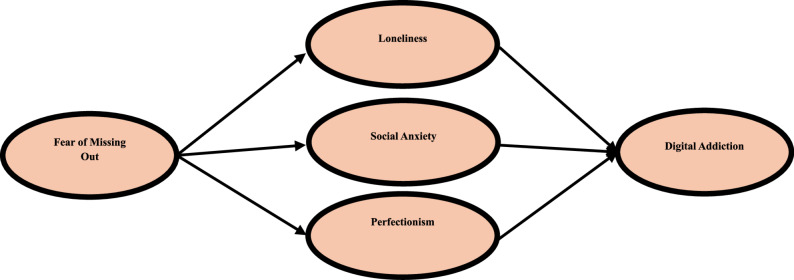
Hypothetical Model of the Study

In this study, which aims to examine the mediating effects of loneliness, social anxiety, and fear of missing out in social contexts on the relationship between perfectionism and digital addiction, one of the quantitative research models-namely, the relational research model-was adopted. Karasar [28] defines the relational research model as one in which it is investigated whether two or more variables change together and to what extent they influence each other. Considering that this study investigates the relationships among perfectionism, digital addiction, loneliness, social anxiety, and fear of missing out in social contexts, this model was deemed appropriate. The mediation model to be tested in the research is shown in Fig. 1.

In the present study, a relational (correlational) research model was employed to examine the associations between fear of missing out (FoMO) and digital addiction, as well as the potential mediating roles of social anxiety, loneliness, and perfectionism in this relationship. Consistent with the theoretical framework, research questions, hypotheses, and statistical analyses, FoMO was conceptualized as the independent (predictor) variable, digital addiction as the dependent (outcome) variable, and social anxiety, loneliness, and perfectionism as parallel mediating variables.

Accordingly, the proposed mediation model examines whether the association between FoMO and digital addiction is statistically accounted for by individual differences in social anxiety, loneliness, and perfectionism. The model was tested using a multiple mediation framework (PROCESS Model 4), in which all mediators were entered simultaneously. Given the cross-sectional and correlational nature of the study design, the specified paths represent theoretical and statistical associations rather than causal or temporal relationships. The hypothesized mediation model tested in the study is presented in Fig. 1.

As shown in Fig. 1, in the research model, the variable of FoMO serves as the independent variable; loneliness, social anxiety, and perfectionism in social contexts serve as mediating variables; and digital addiction serves as the dependent variable.

### Study group

The population of the research consists of individuals aged between 18 and 30 residing in the Turkey. Inclusion criteria for the study required that participants be active users of social media and spend at least two hours daily on social media platforms. It should be clarified that the inclusion criterion in the present study referred specifically to daily social media use rather than total internet use. Participants were required to be active users of social media platforms; however, the variable presented in Table 1 as “duration of internet use” reflects the total amount of time spent online across all activities, including academic work, email communication, and general browsing. Therefore, these two variables capture conceptually different aspects of digital behavior and are not directly comparable. It is also possible for individuals to engage with social media multiple times per day for short durations, which may not be fully reflected in total daily internet use reports. This distinction has been added to avoid potential misinterpretation. Additionally, participants were required to own a smartphone and explicitly report experiencing FoMO in digital environments. To ensure the validity and reliability of the study, certain exclusion criteria were also established. Individuals diagnosed with psychosis, those with a history of cognitive impairment, and those who had been hospitalized in a psychiatric facility for any reason within the past six months were excluded from the study.

Participants were required to self-report experiencing FoMO in digital environments. This criterion was included to ensure that the sample represented individuals who are meaningfully engaged with digital content and thus relevant to the study’s aims. The intention was not to exclude individuals with low FoMO levels, but rather to focus on those who acknowledged awareness of FoMO-related feelings. FoMO levels were still measured continuously using the Fear of Missing Out in Social Contexts Scale, allowing for adequate variance in scores.

A total of 2,372 participants were included in the study. The sample size used in the study (*N* = 2,372) was determined within a planning framework aimed at achieving high statistical power in accordance with the research objectives. Studies examining the relationships among multidimensional psychological variables such as digital addiction, FoMO, social anxiety, loneliness, and perfectionism typically employ complex statistical techniques, including mediation analyses and multivariate regression models. For these models to function reliably, large sample sizes are required to ensure accurate estimation of effect sizes and to enhance the reliability and generalizability of the findings. Accordingly, during the study design phase, a sample size sufficient to detect medium effect sizes (e.g., *r* = .20–0.30) with a 95% confidence level (α = 0.05) and a statistical power of 0.80 was targeted. Although power analysis results indicated that approximately *N* ≈ 300–400 participants would be sufficient to achieve this level of power in multivariate models, the expanded sample size increased statistical power and allowed demographic variables to be included as control variables in the mediation analyses. Therefore, the sample size was substantially expanded. As a result, reaching a total of 2,372 participants enabled the study to meet the following scientific objectives.

Participants were recruited using the convenience sampling method. Convenience sampling is a method in which the researcher collects data from individuals who are most easily accessible [29].As the data in this study were also collected from adult individuals who were easily accessible, the use of this sampling method was deemed appropriate for the research. Sociodemographic characteristics were collected via self-report from the participants. Also, categorical groupings for variables such as anxiety and communication ability were based on participants’ self-reported responses rather than standardized scale cut-offs. For example, participants indicated their perceived anxiety levels and communication abilities, which were then categorized as very low, low, moderate, high, and very high according to their own assessments. This approach allowed us to capture participants’ subjective experiences while providing meaningful groupings for analysis. The sociodemographic characteristics of the participants included in the study are presented in Table 1.

**Table 1:** Sociodemographic Characteristics of the Participants

Variable	Groups	*n*	%
Gender	Female	1417	59,70
	Male	955	40,30
Age	18–20	521	22,00
	21–23	289	12,20
	24–26	471	19,90
	27–30	1091	46,00
Educational Status	Secondary Education (Primary–High)	753	31,70
	Undergraduate–Postgraduate	1619	68,30
Marital Status	Single	1537	64,80
	Married	835	35,20
Socioeconomic Status	Low	374	15,80
	Middle	1912	80,60
	High	86	3,60
General Anxiety Level	Very Low	139	5,90
	Low	479	20,20
	Middle	853	36,00
	High	687	29,00
	Very High	214	9,00
Communication Status	Very Low	19	0,80
	Low	298	12,60
	Middle	869	36,60
	High	949	40,00
	Very High	237	10,00
Receiving Psychological Support	Yes	566	23,90
	No	1806	76,10
Most Frequently Used Digital Device	Mobile phone	2167	91,40
	Computer/Tablet	205	8,60
Purpose of Use	For communication	933	39,30
	For research	190	8,00
	Social sharing	1103	46,50
	Playing games	62	2,60
	Online shopping	30	1,30
	Other	54	2,30
Duration of Internet Use	Less than 1 h	113	4,80
	Between 1–3 h	855	36,00
	Between 3–5 h	659	27,80
	More than 5 h	745	31,40
Frequency of Social Media Use	Once a day	187	7,90
	2–3 times a day	457	19,30
	4–5 times a day	493	20,80
	More than 5 times a day	544	22,90
	Every hour	691	29,10
Frequency of Checking	1–5 times	566	23,90
	6–10 times	592	25,00
	11–15 times	332	14,00
	16–20 times	245	10,30
	More than 21 times	637	26,90
Preferred Social Media Platform	Instagram	1401	59,10
	YouTube	277	11,70
	Twitter (X)	412	17,40
	Facebook	145	6,10
	Other Social Media	137	5,80

“Duration of internet use” refers to total time spent online across all activities and is not limited to social media use

Participant characteristics are presented in Table 1. The sample consisted of 2,372 participants, with a higher proportion of women (59.7%) than men (40.3%). Participants were predominantly young adults, with the largest age group being 27–30 years (46%). Most participants had at least an undergraduate level of education (68.3%) and were single (64.8%). The majority reported a middle socioeconomic status (80.6%). Regarding psychological variables, moderate (36.0%) and high (29.0%) anxiety levels were the most frequently reported. Communication skills were generally perceived as moderate to high. Most participants (76.1%) indicated that they had not previously received professional psychological support. In terms of digital behavior, mobile phones were the primary device used (91.4%). All participants were active users of digital media and social networking platforms. The most common purposes for digital device use were social sharing (46.5%) and communication (39.3%). Daily internet use was typically between one and five hours, although a substantial proportion reported more than five hours of use (31.4%). Social media engagement was frequent, with many participants checking their accounts multiple times per day. Instagram was the most widely used social media platform (59.1%).

Inclusion criteria required participants to own a smartphone, use social media on a daily basis, and self-report experiencing fear of missing out (FoMO) in digital environments. Individuals with a diagnosis of psychotic disorders, cognitive impairment, or recent psychiatric hospitalization were excluded from the study to ensure the reliability of self-reported data.

Detailed sociodemographic characteristics of the participants, including age distribution, gender, educational status, socioeconomic level, patterns of digital device use, duration and frequency of internet and social media use, and preferred platforms, are presented in Table 1. To avoid redundancy, these characteristics are not reiterated in detail in the text.

### Data collection instruments

Data from the participants included in the study were collected through the Sociodemographic Information Form, the Digital Addiction Scale, the Frost Multidimensional Perfectionism Scale, the UCLA Loneliness Scale Short Form-6, the Liebowitz Social Anxiety Scale, and The Fear of Missing Out in Social Contexts Scale.

### Sociodemographic information form

This form was developed by the researcher to systematically gather information on participants’ gender, age, marital status, time spent in digital environments, and the digital platforms they use.

### Digital addiction scale

Developed by Kesici and Tunç [30], this scale consists of a total of 19 items and is structured around five factors: excessive use (items 1–5 / e.g. I find myself checking my digital devices upon dealing with a’work.)), restarting (items 6–8 / e.g. I can not reduced the time I spend with digital devices), disruption of daily life (items 9–12 / e.g. I am unaware of what happens around me while I am dealing with digital devices), mood (items 13–16 / e.g.The environment where I can not use the digital devices bore me ), and difficulty in quitting (items 17–19 /e.g. Even if I live my hause for a short time, I want to take digital devices like phone/tablet with me). Higher scores on the scale indicate an increase in the level of digital addiction. Furthermore, there are no reverse items on the scale. Each item on the scale is rated on a 5-point Likert scale ranging from 1 (Strongly Disagree) to 5 (Strongly Agree). Participants can score between a minimum of 19 and a maximum of 95. Higher scores indicate a higher level of addiction. The Cronbach’s alpha coefficient for internal consistency of the scale was determined to be 0.87 [30]. In the current study, the Cronbach’s alpha value for the total score was calculated as.97.

### Frost multidimensional perfectionism scale

Developed by Frost et al. [20] and adapted into Turkish by Kağan [31], the Frost Multidimensional Perfectionism Scale is used to assess different dimensions of perfectionism. The scale is structured in a 5-point Likert format (1: Strongly Disagree, 5: Strongly Agree) and includes a total of 35 items. It comprises six subscales: “Concern over Mistakes” (items 10, 13, 14, 18, 21, 23, 25, 34- e.g. “When I make a mistake, I feel disappointed.”. ), “Personal Standards” (items 4, 6, 9, 12, 16, 19, 24, 30- e.g. “If I do not set the highest standards for myself, I may become a second-rate person.” ), “Parental Expectations” (items 1, 11, 15, 20, 26- e.g. “My parents’ expectations regarding my future have always been much higher than my own.”), “Parental Criticism” (items 3, 5, 22, 35- e.g. “I never feel as though I can meet my family’s standards.”), “Doubts about Actions” (items 17, 28, 32, 33- e.g. “I tend to fall behind in my work because I do things over and over again.”), and “Organization” (items 2, 7, 8, 27, 29, 31- e.g. “Organization is very important to me.”

). The total score ranges from 35 to 175, with higher scores indicating a higher level of perfectionism. There are no reverse-scored items on the scale. In the reliability analysis of the Turkish version, the Cronbach’s alpha coefficient was calculated as 0.91 [31]. The subscale Cronbach’s alpha coefficients were found to be 0.94 for organization, 0.85 for concern over mistakes, 0.64 for doubts about actions, 0.84 for parental expectations, 0.72 for parental criticism, and 0.79 for personal standards [31]. In the current study, the Cronbach’s alpha value for the total score of the scale was calculated as 0.89.

### UCLA Loneliness Scale – 6-Item Short Form

This scale is a shorter version of the 20-item UCLA Loneliness Scale and consists of 6 items. The UCLA Loneliness Scale – 6-Item Short Form was developed by Wongpakaran et al. [32]. The Turkish adaptation of the scale was conducted by Elbi et al. [33]. The scale assesses participants’ perception of loneliness with six items on a single dimension using a 4-point Likert scale; the response options are ordered as “never,” “rarely,” “sometimes,” and “often.” Scores range between 6 and 24, with higher total scores indicating a greater sense of loneliness. Some example ıtems: “ I feel left out”, “I feel isolated from others”. Since all items are coded in the same direction, the scale does not include any reverse-scored items. In the Turkish adaptation study, the scale demonstrated a strong internal consistency coefficient (Cronbach’s alpha = 0.884) and showed a homogeneous structure with item-total correlations ranging from 0.742 to 0.840. The variance explained by the single dimension of the scale was calculated as 63.41%. In the present study, the Cronbach’s alpha coefficient for the total score of the scale was calculated as 0.78.

### Liebowitz Social Anxiety Scale (LSAS-SR)

The Liebowitz Social Anxiety Scale – Self-Report (LSAS-SR) was originally developed by Michael R. Liebowitz in 1987, and its Turkish validity and reliability study was conducted by Soykan, Özgüven, and Gençöz [34]. The LSAS-SR is a self-report instrument designed to assess fear and avoidance related to social interaction and performance situations in individuals with social anxiety. The scale consists of 24 items, of which 11 assess social interaction situations and 13 assess performance situations, forming two subscales: fear/anxiety and avoidance. Participants rate each situation by responding to questions such as “How much fear or anxiety do you feel in this situation?” and “How often do you avoid this situation?” using Likert-type response options. The total score is obtained by summing the fear and avoidance ratings across all items. Higher scores indicate greater levels of social anxiety and avoidance behaviors. In the Turkish adaptation study, the internal consistency coefficient was reported as 0.98. Convergent validity was supported by significant positive correlations with the Beck Anxiety Inventory among individuals diagnosed with social phobia, while discriminant validity was demonstrated by the absence of a significant association in the general sample, indicating that the scale specifically measures social anxiety. Criterion validity analyses further showed that the LSAS-SR successfully distinguished individuals with social phobia from both non-clinical individuals and those with other anxiety disorders. Overall, the Turkish version was found to be a reliable and valid instrument for assessing social anxiety. In the present study, the LSAS-SR demonstrated excellent psychometric properties: Cronbach’s alpha (α) = 0.99, McDonald’s Omega (ω) = 0.99, Average Variance Extracted (AVE) = 0.74, Composite Reliability (CR) = 0.95, and Heterotrait–Monotrait ratio of correlations (HTMT) = 0.93.

### Fear of missing out in social contexts scale

This scale was developed by Przybylski et al. [2] and adapted into Turkish by Gökler et al. [35]. It is a 10-item instrument using a 5-point Likert-type scale. Participants complete the scale based on their self-assessments related to their own lives. Each item is rated between 1 (not at all true of me) and 5 (completely true of me) e.g. “ I get anxious when I don’t know what my friends are up to.”, “ I fear my friends have more rewarding experiences than me.”The total score ranges from 10 to 50, and there is no predetermined cut-off score. A higher total score indicates a more intense fear of missing out. According to the results of factor analysis, the scale has a unidimensional structure, and item factor loadings range from 0.36 to 0.77. The Cronbach’s alpha internal consistency coefficient of the scale was found to be 0.81, and the test-retest reliability coefficient was also 0.81. In the current study, the scale demonstrated high reliability and validity, with Cronbach’s alpha (α) = 0.95, McDonald’s Omega (ω) = 0.98, Average Variance Extracted (AVE) = 0.68, Composite Reliability (CR) = 0.95, and Heterotrait-Monotrait ratio of correlations (HTMT) = 0.90.

In the present study, both the FoMO and social anxiety scales demonstrated excellent reliability and validity. The FoMO scale showed Cronbach’s alpha (α) = 0.95, McDonald’s Omega (ω) = 0.98, Average Variance Extracted (AVE) = 0.68, Composite Reliability (CR) = 0.95, and Heterotrait-Monotrait ratio of correlations (HTMT) = 0.90, whereas the social anxiety scale exhibited Cronbach’s alpha (α) = 0.99, McDonald’s Omega (ω) = 0.99, AVE = 0.74, CR = 0.95, and HTMT = 0.93. These indices indicate that both scales are highly reliable and the items consistently reflect their intended constructs. Convergent validity is supported for both instruments, as evidenced by AVE values above the recommended threshold of 0.50 and CR values exceeding 0.70. Despite these positive indicators, the HTMT values for both scales are slightly above the commonly recommended threshold of 0.85–0.90, suggesting potential overlap between the constructs, which is theoretically plausible given that FoMO and social anxiety are conceptually related. The elevated HTMT highlights that individuals with high FoMO tendencies may also experience heightened social anxiety, reflecting the interconnected nature of concerns about missing social experiences and social evaluation. While discriminant validity is generally supported, these findings call for careful interpretation of the constructs as fully distinct. Future research could consider model refinements, such as item parceling, bifactor modeling, or examining cross-loadings, to better delineate the boundaries between FoMO and social anxiety. Overall, the results confirm that both scales are psychometrically robust and suitable for research in digital and social contexts, while also emphasizing the importance of considering construct overlap when examining the interplay between FoMO and social anxiety.

### Procedure

The study commenced following the approval of the Ethics Committee of Development University (date and approval number to be specified). Permissions for the use of the scales employed in the study were obtained via email from the original authors or the researchers responsible for the Turkish adaptation prior to the data collection process. The research data were collected between May and July 2026. Participants were recruited via [online invitations shared on social media platforms (such as Whatsapp, Instagram, Facebook…etc) and institutional mailing lists]. Interested individuals accessed the online survey through a provided link. The scales were administered online using the ‘Google Forms’ platform. Participants were presented, in order, with the Digital Addiction Scale, the Frost Multidimensional Perfectionism Scale, the UCLA Loneliness Scale Short Form-6, the Liebowitz Social Anxiety Scale, and the Fear of Missing Out in Social Contexts Scale. Participants completed the scales online using their own devices, including smartphones, tablets, or laptops. They accessed the survey via the provided online link and submitted their responses directly to the researchers. There were no missing data in the study. To ensure complete responses, the online survey was set to a forced-response mode, requiring participants to answer each item before proceeding to the next question. This procedure prevented any missing data across all administered scales. Before administering the scales, informed consent was obtained from the participants, emphasizing that the research was based on voluntary participation; care was taken to ensure participant confidentiality during data collection and storage processes.

### Data analysis

SPSS 25.0 statistical software package was used for data analysis. Prior to conducting the analysis, the presence of outliers and extreme values was examined using Cook’s distance and Mahalanobis distance. As a result of the Mahalanobis and Cook’s distance calculations, no outliers were identified. Also, to assess potential common method bias (CMB), a Harman single-factor test was conducted. Results indicated that a single factor accounted for 35% of the total variance, which is below the commonly accepted threshold of 50% [36], suggesting that common method bias is unlikely to be a serious concern in the present study. Subsequently, skewness and kurtosis values were examined to determine whether the data set followed a normal distribution. Information related to the normality distribution of the research data is presented in Table 2.

**Table 2:** Descriptive Statistics

Variable Name	Mean	SD	Minimum	Maximum	Skewness	Kurtosis
Digital Addiction	2.80	1.03	1	5	0.293	-0.872
Perfectionism	103.97	23.19	52	160	0.202	-0.805
Fear of Missing Out (FoMO)	24.74	11.10	10	50	0.756	-0.647
Loneliness	13.59	4.88	6	24	0.191	-0.967
Social Anxiety	94.99	36.92	48	192	0.736	-0.635

Tabachnick and Fidell [37] suggest that if the skewness and kurtosis values of scale scores fall between − 1.5 and + 1.5, the distribution can be considered normal. In the present study, since the skewness and kurtosis values of the research variables fall within the − 1.5 to + 1.5 range, it can be stated that the data set demonstrates a normal distribution. Accordingly, as the scale data are normally distributed, parametric tests were used in the analysis of the data. To examine the relationships among the research variables, Pearson correlation analysis was conducted. Additionally, mediation analysis was performed using the PROCESS macro based on Hayes’ Model 4, with a 95% confidence interval and 5,000 bootstrap samples. It should be noted that the coefficients reported in the mediation analyses are unstandardized regression coefficients, as provided by the default settings of the PROCESS macro. Accordingly, their magnitude is influenced by the measurement scales and score ranges of the variables, meaning that variables with wider score ranges may yield larger coefficient values. For this reason, the coefficients should be interpreted in relation to the scale metrics rather than their absolute size. All variables were entered into the analyses in their original form, and no mean-centering or standardization procedures were applied. In addition, gender and age were included as covariates to control for their potential confounding effects; gender was coded as a binary variable (0 = male, 1 = female), and age was entered as a continuous variable.

Before proceeding with the mediation analysis, the basic assumptions of regression analysis, namely multicollinearity and autocorrelation, were examined. The Variance Inflation Factor (VIF) and tolerance values were used to assess whether multicollinearity existed among the independent variables. In the literature, it is recommended that VIF values be below 10 and tolerance values be above 0.20 [38]. In this study, the VIF values for the independent and mediating variables were found to be less than 10, and the tolerance values were greater than 0.20. This finding indicates that there is no multicollinearity problem.

In addition, assessing whether there is a correlation among error terms is another important assumption of regression analysis. For this purpose, the Durbin-Watson test was employed. The Durbin-Watson coefficient tests whether there is serial correlation among the error terms, and a significant coefficient indicates the presence of autocorrelation among the residuals [37]. A Durbin-Watson value between 1.5 and 2.5 indicates that there is no autocorrelation [39]. In this study, the Durbin-Watson value was calculated as 1.838, which indicates that there is no autocorrelation among the error terms. (Table 3).

**Table 3:** Pearson Correlation Analysis Findings

		1	2	3	4
1. Digital Addiction	*r* (*r*^2^)	1			
2. Perfectionism	r (r^2^)	− 0.070^**^ (0.01)	1		
3. Loneliness	r (r^2^)	− 0.041* (0.00)	− 0.055** (0.00)	1	
4. Social Anxiety	r (r^2^)	0.685** (0.47)	0.115** (0.01)	0.054** (0.00)	1
5. Fear of Missing Out (FoMO)	r (r^2^)	0.748** (0.56)	− 0.122** (0.02)	0.027 (0.00)	0.792** (0.63)

*p<.05, **p<.001; Note: r² values indicate the proportion of shared variance between the variables, with 0.01 considered small, 0.09 medium, and 0.25 large according to Cohen’s (1988) guidelines

### Findings

This section presents the results of the statistical analyses conducted to test the research hypotheses.

To test the direction and significance of the relationships among the research variables, Pearson correlation analysis was conducted. Also, The effect sizes of the correlations among the variables were calculated using the coefficient of determination (r²).

Digital Addiction showed a small negative correlation with perfectionism (*r* = -.07, r² = 0.01, *p* < .01) and with loneliness (*r* = -.041, r² = 0.00, *p* < .05), and a strong positive correlation with social anxiety (*r* = .685, r² = 0.47, *p* < .01) andFoMO; *r* = .748, r² = 0.56, *p* < .01).

Perfectionism was weakly correlated with social anxiety (*r* = .115, r² = 0.01, *p* < .01) and negatively with FoMO (*r* = -.122, r² = 0.02, *p* < .01). Loneliness showed negligible correlations with social anxiety (*r* = .054, r² = 0.00, *p* < .01) and FoMO (*r* = .027, r² = 0.00, ns). Finally, social anxiety was strongly positively associated with FoMO (*r* = .792, r² = 0.63, *p* < .01). (Table 4).

**Table 4:** Mediation Analysis Findings

Type of Effect	Pathway	Effect Coefficient	t	*p*	Bootstrap SE		LLCI	ULCI	*R*²
Total Effect	FoMO → Digital Addiction	0.0700	52.44	< 0.001	0.0013		0.0673	0.0726	0.56
Direct Effect	FoMO → Digital Addiction	0.0520	25.47	< 0.001	0.0020		0.0480	0.0560	0.58
Indirect Effect	FoMO → Loneliness → Digital Addiction	0.0000	-	-	0.0000	-0.0001		0.0001	-
	FoMO → Social Anxiety → Digital Addiction	0.0181	-	-	0.0019	0.0145		0.0219	-
	FoMO → Perfectionism → Digital Addiction	-0.0002	-	-	0.0001	-0.0005		0.0001	-
Total Indirect Effect	Sum of all mediators	0.0179	-	-	0.0019		0.0142	0.0218	-1.

Covariates: Gender and Age. Bootstrap samples = 5,000. CI = 95%; Coefficients are unstandardized and depend on the scale ranges of the variables

A parallel multiple mediation analysis was conducted using PROCESS Model 4 (5,000 bootstrap samples) to examine whether loneliness, social anxiety, and perfectionism mediated the relationship between fear of missing out (FoMO) and digital addiction, while controlling for gender and age as covariates. Results showed that FoMO had a significant total effect on digital addiction (b = 0.070, SE = 0.001, t = 52.44, *p* < .001, 95% CI [0.067, 0.073]). When the mediators were included in the model, the direct effect of FoMO decreased but remained significant (b = 0.052, SE = 0.002, t = 25.47, *p* < .001, 95% CI [0.048, 0.056]), indicating partial mediation.

The total indirect effect was significant (b = 0.018, BootSE = 0.002, 95% CI [0.014, 0.022]). Examination of specific indirect effects revealed that only the indirect pathway through social anxiety was significant (b = 0.018, BootSE = 0.002, 95% CI [0.015, 0.022]). In contrast, the indirect effects through loneliness (b = 0.000, 95% CI [− 0.0001, 0.0001]) and perfectionism (b = − 0.0002, 95% CI [− 0.0005, 0.0001]) were not significant, as their confidence intervals included zero. The overall model explained 58% of the variance in digital addiction (R² = 0.584).

Regarding the covariates, results from the final outcome model indicated that gender (b = 0.053, SE = 0.030, t = 1.80, *p* = .073) and age (b = 0.002, SE = 0.012, t = 0.13, *p* = .895) were not significant predictors of digital addiction. However, analyses of the mediator models showed that age had a significant positive effect on loneliness (b = 0.271, *p* = .001). Gender significantly predicted both social anxiety and perfectionism (respectively, b = − 3.95, *p* < .001; b = 6.82, *p* < .001). Additionally, age had significant negative effects on social anxiety (b = − 0.86, *p* = .027) and perfectionism (b = − 1.04, *p* = .008). These findings suggest that demographic variables may play a role at the level of the mediators, but they do not substantially account for the direct association between FoMO and digital addiction.

Examination of the individual mediation paths showed that FoMO significantly predicted social anxiety (a-path), and social anxiety significantly predicted digital addiction (b-path). However, the a- and b-paths associated with loneliness and perfectionism were not statistically significant. These results suggest that the indirect effect of FoMO on digital addiction operates primarily through social anxiety.

Because the present study is based on cross-sectional data, the temporal ordering between fear of missing out (FoMO) and digital addiction cannot be established with certainty. Although the primary model was theoretically grounded in the assumption that FoMO is positively associated with digital addiction.digital addiction, alternative directional pathways are also plausible. For example, excessive engagement in digital environments may heighten individuals’ concerns about missing out on social experiences, thereby increasing FoMO, or the relationship between these constructs may be reciprocal in nature. To strengthen the robustness of the findings and address this limitation, an alternative (reverse) mediation model was also tested in which digital addiction was specified as the predictor and FoMO as the outcome variable. The results of this additional analysis are reported to provide a more comprehensive understanding of the potential directional dynamics between these variables. (Table 5).

**Table 5:** Reverse Mediation Analysis Findings

Type of Effect	Pathway	Effect Coefficient	t	*p*	Bootstrap SE		LLCI	ULCI	*R*²
Total Effect	Digital Addiction → FoMO	76.875	52.44	< 0.001	-		74.001	79.750	0.58
Direct Effect	Digital Addiction → FoMO	41.425	25.47	< 0.001	-		38.235	44.615	0.71
Indirect Effect	Digital Addiction → Loneliness → FoMO	-0.0066	-	-	0.0065	-0.0223		0.0030	-
	Digital Addiction → Social Anxiety → FoMO	35.385	-	-	0.1558	32.377		38.602	-
	Digital Addiction → Perfectionism → FoMO	0.0132	-	-	0.0084	0.0001		0.0323	-
Total Indirect Effect	Sum of all mediators	35.450	-	-	0.1562		32.440	38.620	-

Covariates: Gender and Age. Bootstrap samples = 5,000. CI = 95%; Note 2. Coefficients are unstandardized and depend on the scale ranges of the variables

To examine the alternative directional hypothesis that FoMO may be an outcome of digital addiction, a parallel multiple mediation analysis was conducted in which digital addiction was specified as the predictor and FoMO as the outcome variable. Loneliness, social anxiety, and perfectionism were included as mediators, while gender and age were entered as covariates. Analyses were performed using PROCESS Model 4 with 5,000 bootstrap samples.

Results indicated that the total effect of digital addiction on FoMO was significant (b = 7.69, SE = 0.15, t = 52.44, *p* < .001, 95% CI [7.40, 7.98]). When the mediators were included in the model, the direct effect of digital addiction on FoMO decreased but remained significant (b = 4.14, SE = 0.16, t = 25.47, *p* < .001, 95% CI [3.82, 4.46]), indicating partial mediation.

The total indirect effect was significant (b = 3.55, BootSE = 0.16, 95% CI [3.24, 3.86]). Examination of specific indirect pathways showed that the effect of digital addiction on FoMO was primarily mediated by social anxiety (b = 3.54, BootSE = 0.16, 95% CI [3.24, 3.86]). In addition, the indirect effect through perfectionism was small but statistically significant (b = 0.01, BootSE = 0.01, 95% CI [0.0001, 0.03]). In contrast, the indirect effect through loneliness was not significant (b = − 0.007, 95% CI [− 0.022, 0.003]), as the confidence interval included zero. The overall model, including mediators and covariates, explained 71% of the variance in FoMO (R² = 0.713).

Regarding the covariates, results from the final model indicated that gender significantly predicted FoMO (b = − 1.35, SE = 0.26, *p* < .001) and age also had a significant negative effect on FoMO (b = − 0.26, SE = 0.10, *p* = .012). Analyses of the mediator models further showed that age positively predicted loneliness, and both gender and age significantly predicted levels of social anxiety and perfectionism. These findings suggest that the association between digital addiction and FoMO is largely explained through social anxiety, while demographic variables play a role both in FoMO and in several of the mediating variables.

## Discussion

The present study aimed to clarify the psychological processes underlying the association between FoMO and digital addiction. Rather than representing a simple direct link, the findings suggest that this relationship is embedded within a broader network of social–emotional mechanisms, with social anxiety emerging as a central explanatory factor. Causal interpretations are beyond the scope of the present design and are discussed in the limitations section.

FoMO has been conceptualized as a persistent concern about missing rewarding social experiences Przybylski et al., [2]. From this perspective, digital environments provide immediate and continuous access to social information, making them particularly attractive for individuals high in FoMO. However, this engagement may not be driven solely by curiosity or social interest; it may also function as a way to regulate anxiety related to social evaluation and exclusion. This interpretation aligns with cognitive–behavioral models of social anxiety, whichtmth propose that individuals who fear negative evaluation tend to engage in safety behaviors and excessive monitoring of social cues Clark & Wells, [43, 44]. Digital platforms, with their opportunities for asynchronous communication and controlled self-presentation, may therefore serve as compensatory spaces for socially anxious individuals.

The findings are also consistent with broader models of problematic internet use, such as the I-PACE framework, which emphasizes the interaction of affective vulnerabilities and cognitive processes in the development of maladaptive digital behaviors Brand et al., [49]. Within this framework, FoMO can be understood as a cognitively driven concern about social connectedness, while social anxiety represents an affective vulnerability that intensifies the urge to seek reassurance and social monitoring through digital media. Together, these processes may foster patterns of excessive or compulsive engagement with digital platforms.

In contrast, loneliness and perfectionism did not appear to play a central intermediary role in the FoMO–digital addiction link. Although loneliness has often been associated with increased digital media use, it primarily reflects perceived deficits in social connectedness rather than heightened sensitivity to social comparison and evaluation Oberst et al., [51]. FoMO, by definition, is more closely tied to concerns about others’ activities and social inclusion than to general feelings of isolation. Similarly, perfectionism is typically linked to self-evaluative standards and performance-related concerns. While such traits may shape online behavior in specific contexts, they may be less directly connected to the socially comparative and immediacy-driven processes that characterize FoMO. These distinctions suggest that FoMO-related digital engagement is more strongly rooted in social evaluative anxiety than in broader experiences of isolation or achievement-oriented self-criticism.

The alternative directional model further highlights the possibility that the relationship between FoMO and digital addiction may be reciprocal rather than strictly unidirectional. Intensive involvement in digital environments increases exposure to others’ activities, social comparisons, and expectations of constant availability, all of which may amplify FoMO over time Blackwell et al., [48]. This reciprocal perspective aligns with emerging views that problematic digital engagement both reflects and reinforces existing psychological vulnerabilities. The consistent involvement of social anxiety across directional models underscores its role as a key mechanism linking social–emotional concerns with digital behavior Wegmann & Brand, [49, 50].

Demographic variables were not central in explaining the FoMO–digital addiction relationship, suggesting that this association is driven more strongly by psychological processes than by age or gender alone. The present study did not test moderation effects; rather, demographic variables were statistically controlled to reduce potential confounding influences. While demographic factors may influence levels of social anxiety or perfectionism, the core dynamics connecting FoMO and digital addiction appear to operate primarily through socio-emotional regulation mechanisms (Brand et al., [49].

Overall, the study contributes to the literature by framing digital addiction not merely as a consequence of technological exposure, but as a behavior deeply embedded in social–emotional regulation processes. FoMO appears to increase vulnerability to problematic digital engagement particularly when combined with heightened social anxiety, pointing to the importance of addressing underlying social-evaluative concerns in both prevention and intervention efforts. Future longitudinal and experimental studies are needed to clarify the temporal ordering and reciprocal influences among these constructs.

### Conclusion, recommendations and limitations

When the research findings are evaluated holistically, it is observed that there are significant relationships among digital addiction, social anxiety, and fear of missing out in social contexts. The results indicate that the extent to which individuals’ basic psychological needs are met shapes their behavior regarding digital environment usage. In line with theoretical frameworks such as the Self-Determination Theory and the Fundamental Interpersonal Needs Theory, it is understood that when individuals’ needs for establishing relationships, belonging, and feeling competent are not met, they tend to spend more time on social media, which may increase digital addiction. Moreover, while social anxiety leads individuals to avoid social environments, their tendency to compensate for this social deficiency through digital interactions increases their level of addiction and simultaneously strengthens the fear of missing out in social contexts. In this regard, it has been observed that social anxiety plays a partial mediating role in the relationship between fear of missing out in social contexts and digital addiction. On the other hand, it is noteworthy that the variables of loneliness and perfectionism do not show a mediating role in this relationship. In particular, the negative relationships identified between perfectionism and both digital addiction and fear of missing out in social contexts suggest that perfectionist individuals tend to act more deliberately, in a controlled and purposeful manner in digital environments. Similarly, the negative relationship between loneliness and digital addiction reveals that while digital interactions may temporarily reduce feelings of loneliness, they do not provide a permanent solution in the long term. As a result, the research demonstrates that digital addiction is closely related to psychosocial conditions such as social anxiety and fear of missing out on social developments; and that individuals’ use of digital media is not only rooted in technological factors but also grounded in psychological and social dimensions.

It should be noted that the present study employed convenience sampling, which may limit the generalizability of the findings to broader populations. In addition, the inclusion criterion requiring participants to explicitly report experiencing FoMO in digital environments may have inflated the observed associations among the variables, as the sample may over-represent individuals with higher FoMO levels. Consequently, these methodological considerations suggest that the results should be interpreted with caution, and future research should aim to replicate the findings using more diverse and representative samples.

Another limitation of the present study is the lack of detailed information regarding participants’ prior mental health conditions. Although individuals with psychotic disorders, cognitive impairment, or recent psychiatric hospitalization were excluded, more comprehensive clinical history data were not collected. Therefore, it is possible that some of the elevated scores observed in the study may be partially attributable to pre-existing psychological conditions. This limitation should be considered when interpreting the findings and the generalizability of the results.

In line with the findings obtained, it is possible to offer several recommendations to future researchers and clinicians. First and foremost, in understanding one of today’s prevalent issues such as digital addiction, the influence of psychosocial variables like social anxiety and in social contexts should be taken into account.; It is important that future research be conducted on larger and more diverse samples in order to contribute to a deeper understanding of the complex relationships between social anxiety, loneliness, and perfectionism. In particular, there is a need for longitudinal studies examining the effects of digital addiction and FoMO. Such studies will allow for a better understanding of the temporal changes in individuals’ social relationships and emotional states, as well as an exploration of the dynamics of factors associated with social anxiety [8]. in particular, variables influencing the development of digital addiction and mediating factors should be examined in detail. One of the limitation concerns the inclusion criterion requiring participants to self-report FoMO experiences. While this ensured conceptual relevance to the study’s objectives, it may have slightly restricted the variability of FoMO scores in the sample. Future studies could employ random sampling procedures to obtain a broader distribution of FoMO levels.

In terms of clinical practice, when planning interventions targeting digital media use among individuals with high levels of social anxiety, structured social skills training programs, cognitive-behavioral therapies, and mindfulness-based approaches that support the individual’s basic psychological needs (relatedness, belongingness, competence) may be preferred. Moreover, in interventions aimed at preventing digital addiction, it is important to develop psychoeducational programs designed to reduce individuals’ levels of fear of missing out in social contexts.

The present study aimed to examine the associations between fear of missing out (FoMO) and digital addiction, and to explore the potential explanatory roles of social anxiety, loneliness, and perfectionism within this relationship. Rather than reiterating the statistical findings, the following discussion focuses on the theoretical meaning of the results, their consistency with previous research, and their broader implications for understanding problematic digital engagement among young adults.

Consistent with existing literature, the findings indicate a strong association between FoMO and digital addiction. This relationship aligns with theoretical accounts suggesting that FoMO reflects heightened sensitivity to social exclusion, comparison, and belonging-related concerns in digitally mediated environments. Individuals who experience persistent concerns about missing social experiences may engage more intensively with digital platforms as a way of monitoring social information and maintaining perceived inclusion. Importantly, this association should be understood as relational rather than causal, yet it remains theoretically meaningful within contemporary models of digital media use.

A central contribution of the present study lies in highlighting social anxiety as a key psychological factor associated with the FoMO–digital addiction link. The results suggest that individuals who experience elevated FoMO also tend to report higher levels of social anxiety, which is in turn associated with greater problematic digital engagement. This pattern is consistent with cognitive–behavioral models of social anxiety, which emphasize heightened self-monitoring, fear of negative evaluation, and reliance on safety behaviors. Digital environments may function as compensatory spaces in which socially anxious individuals can regulate interpersonal discomfort while maintaining a sense of social connection, thereby reinforcing excessive use.

From a theoretical perspective, these findings are also compatible with Self-Determination Theory. FoMO can be conceptualized as an affective signal of unmet relatedness needs, and social anxiety may further intensify concerns about social evaluation and exclusion. Together, these processes may be associated with increased engagement in digital contexts where social interaction is more controllable and less immediately threatening. In this sense, social anxiety appears to play a particularly salient explanatory role in understanding why FoMO is linked to problematic digital use.

In contrast, loneliness and perfectionism did not emerge as explanatory variables within the proposed mediation framework. Although both constructs have been associated with FoMO and digital media use in previous studies, the present findings suggest that they may function more as background vulnerabilities or contextual characteristics rather than central mechanisms. Loneliness, for example, may be intermittently alleviated through digital interaction without necessarily accounting for problematic usage patterns. Similarly, perfectionism-especially when considered as a broad trait-may relate to digital behavior in more complex or domain-specific ways that are not fully captured within a general mediation model.

When compared with prior research, the present findings converge with studies emphasizing the role of anxiety-related processes in problematic digital engagement, while extending this literature by clarifying the relative importance of social anxiety over other psychosocial factors. The use of a large and heterogeneous sample further strengthens the robustness of these associations and supports their relevance for understanding digital behavior in emerging adulthood, a developmental period marked by heightened sensitivity to peer evaluation and social belonging.

From a practical standpoint, the findings underscore the importance of addressing FoMO-related cognitions and social anxiety symptoms in interventions targeting digital addiction. Preventive and clinical efforts may benefit from focusing not only on regulating digital use behaviors, but also on reducing excessive social monitoring, fear of evaluation, and reassurance-seeking tendencies. Interventions grounded in cognitive–behavioral and mindfulness-based approaches may be particularly well-suited to addressing these underlying processes.

Overall, the present study contributes to the growing body of research on digital addiction by emphasizing the interconnected roles of FoMO and social anxiety within contemporary digital environments. By shifting the focus from purely behavioral explanations to psychosocial and motivational processes, the findings offer a more nuanced understanding of problematic digital engagement and highlight important directions for future research and intervention.

## Supplementary Information

Supplementary Material 1.

## Data Availability

Data for this study are available from the corresponding author upon reasonable request.
