# Supplementary material for: The mediating role of social anxiety, loneliness and perfectionism in the relationship between fear of missing out (FoMO) and digital addiction
Source: BMC Psychol. 2026 Apr 21;14:866. doi: 10.1186/s40359-026-04489-3 (PMC13267220; doi:10.1186/s40359-026-04489-3)
Supplement: Supplementary file 1 — Supplementary Material 1. [file 40359_2026_4489_MOESM1_ESM.pdf]

T.C.
TOROS ÜNİVERSİTESİ
BİLİMSEL ARAŞTIRMA VE YAYIN ETİK KURULU ONAY BELGESİ

Toros Üniversitesi Bilimsel Araştırma ve Yayın Etik Kuruluna başvuran Toros Üniversitesi İ.İ.S.B.F. Psikoloji Bölümü Dr. Öğr. Üyesi Gülşen FİLAZOĞLU ÇOKLUK'un **"Dijital Bağımlılık ve FOMO Sosyal Anksiyete ile İlişkisinin Çözülmesi ve Etkili Müdahale Yolları"** adlı çalışması kurulumuz tarafından incelenmiş ve;

Etik Yönden uygun bulunmuştur.	✓
Etik Yönden Geliştirilmesi gerekmektedir.	
Etik Yönden Uygun Bulunmamıştır	

Toros Üniversitesi Bilimsel Araştırma ve Yayın Etik kurul Kararı	
Başvuru Formunun Etik Kurula Ulaştığı Tarih	20.05.2025
Etik kurul Karar Toplantı Tarihi ve Karar No	22.05.2025-99
Yer	Toros Üniversitesi
Katılımcılar	Formda imzası bulunan üyelerimiz toplantıya katılmıştır.

KURUL BAŞKANI VE ÜYELER:

Prof. Dr. Tunay KÖKSAL	Başkan	
Prof. Dr. Köksal HAZIR	Başkan Yrd.	
Prof. Dr. Fügen ÖZCANARSLAN	Üye	
Prof. Dr. Ünsal YETİM	Üye	
Prof. Dr. Sera Yeşim AKSAN	Üye	
Prof. Dr. Yüksel ÖZDEMİR	Üye	
Prof. Dr. Mehmet ÇAKIROĞLU	Üye	

Açıklama:
